# The Water Framework Directive’s protection of groundwater-dependent terrestrial ecosystems

**DOI:** 10.1007/s13280-024-02108-2

**Published:** 2024-12-14

**Authors:** Lasse Baaner, Helle Tegner Anker, Rasmus Ejrnæs

**Affiliations:** 1 Institut for Fødevare- og Ressourceøkonomi, Københavns Universitet, Rolighedsvej 23, 1958 Frederiksberg C, Denmark; 2 Department of Ecoscience, C. F. Møllers Allé 8, Bldg 1110, 8000 Aarhus C, Denmark

**Keywords:** Biodiversity, Drainage, Ecosystem management, Groundwater-dependent terrestrial ecosystems, Water table, Wetlands

## Abstract

The Water Framework Directive protects groundwater-dependent terrestrial ecosystems, but its concepts and definitions remain unclear. This paper aims to clarify the margin of discretion for the Member States, by applying a cross-disciplinary legal and biological analysis. We conclude that description of the protected ecosystems must include at least key components and processes and be based on a number of well-known groundwater-dependent habitats, but not restricted to habitats fed entirely by groundwater. We argue that the potential harm to terrestrial ecosystems by lowering the groundwater table should include the impact of both water abstraction and drainage, and, despite the discretion regarding scale, we recommend basing assessments and protection at a landscape-scale that aligns with the scale of bodies of groundwater, which typically includes a range of habitats in various ecological conditions.

## Introduction

The Water Framework Directive^1^ (WFD) protects Europe's groundwater and surface water, so that the water requirements of both people and nature may be met. One of the natural elements whose water requirements are protected by the WFD is terrestrial ecosystems. This is apparent from the purpose in Article 1 and is also expressed in recital 23 of the preamble.

The WFD protects groundwater-dependent terrestrial ecosystems (GWDTE) by stating in Annex V section 2.1 that good quantitative status of bodies of groundwater requires there to be no significant damage to terrestrial ecosystems directly associated with a body of groundwater as a result of anthropogenic changes to the groundwater table. Correspondingly good chemical status requires that the concentrations of pollutants in the groundwater do not cause significant damage to terrestrial ecosystems as stated in Annex V section 2.3. The protection of all Europe’s groundwater-dependent terrestrial ecosystems against significant damage related to their water needs is a part of the European Union’s protection of biodiversity, though this is somewhat overlooked.

To comply with the foregoing provisions, Member States must identify the scope of the terms ‘groundwater-dependent terrestrial ecosystem’, ‘associated bodies of groundwater’, and ‘significant damage’. Several EU Member States have applied a step-by-step process to identify and assess groundwater-dependent terrestrial ecosystems as discussed by Whiteman et al. (2010) and Terasmaa et al. (2020); however, the scope of the legal provisions and the margin of discretion left to the Member States have not been thoroughly examined and settled. In the following, we argue that basic questions pertaining to the definition and identification of groundwater-dependent terrestrial ecosystems and to significant damage need to be answered prior to the development of assessment methods.

‘Groundwater-dependent terrestrial ecosystems’ is a legal concept, created and defined by EU law, but, like ‘favourable conservation status’, discussed by Epstein (Epstein 2016), and ‘good ecological status’, discussed by Josefsson (2015), it makes sense only when closely linked to fundamental ecological concepts. As Epstein points out, the distinction between legal, ecological, and even political concepts is not always clear. This article takes a combined legal and ecological approach similar to Epstein’s and Josefsson’s and discusses two crucial questions concerning the WFD protection of groundwater-dependent terrestrial ecosystems:

1. What qualifies as a groundwater-dependent terrestrial ecosystem in the sense of WFD, regarding scope, scale, and dependency on groundwater, and what is the Member States’ margin of discretion regarding these?
2. What must be understood as significant damage to terrestrial ecosystems, and how could such damage be assessed?

The answers to these questions form the basis for our discussion of the potential pitfalls EU Member States may encounter, and their management options when they pursue the Directive’s objective of protecting groundwater-dependent terrestrial ecosystems.

## Ecosystems, scale, and groundwater dependency?

### The scope of the word ‘ecosystems’ in the WFD

The Water Framework Directive is often emphasized as based on an ecosystem approach, in contrast to the older water directives.^2^ The Directive addresses several types of ecosystems: aquatic ecosystems, and also wetlands and water-dependent terrestrial ecosystems.

Aquatic ecosystems are included in the Directive's definition of ecological status for surface water in Article 2(21). Here, reference is made to the classification in Annex V, that is, the classification used to assess the condition of surface bodies of water. Therefore, aquatic ecosystems in the sense of the Directive are probably limited to ecosystems that may be described through the Directive's classification system of surface water. It is different with wetlands and terrestrial ecosystems: no classification system is provided for these. The selection of components, functions, and processes to be included in the description of a terrestrial groundwater-dependent ecosystem is left to the Member States.

Aquatic ecosystems, which are the Directive's main object of protection, are not limited to what may be called wild nature or minimally affected natural ecosystems. Aquatic ecosystems, in sense used by the WFD, include both artificial and highly modified bodies of water. This speaks for a correspondingly broad concept of terrestrial ecosystems dependent on groundwater, including as an example cultivated agricultural areas and urban green spaces. On the other hand, the definition of *good status* for aquatic ecosystems is premised on its being only slightly affected by human activity, compared to pristine condition, and special provisions have been established in article 4 for artificial and heavily modified bodies of water. There is also no obligation to identify terrestrial groundwater-dependent ecosystems that have been lost as a result of changes in the landscape and due to land use. This speaks of a narrower scope for the term ‘terrestrial ecosystem’, for example, one that includes only ecosystems with significant presence of sites with relatively intact natural processes and ecosystems, where the protection of biodiversity is of a particular concern.

From the WFD Article 2(21) definition of ecological status, it follows that an ecosystem must be characterized at least by its *structure* and *functioning*. These are legal terms and may not align fully with their usage in ecosystem ecology (e.g. Cummins 1974).

‘Ecosystem structures’ generally covers the distribution and arrangement, or composition, of ecosystem components or elements. Components or elements are the separate entities of the system: abiotic elements, such as bodies of water, layers of soil or underground, nutrient pools, and biotic elements, such as individuals, populations, or organic substrates. Over time, if left undisturbed, ecosystems will often increase in complexity and diversity of components, a phenomenon coined as *expansion* by Brunbjerg et al. (2017). However, ‘ecosystem structures’, and ‘ecosystem components’ or ‘elements’ are also used interchangeably (Haines-Young and Potschin 2010).

The term ‘ecosystem function’ is very general and partly ambiguous, as it is used by some scholars as a synonym for ‘ecosystem processes; (Wallace 2007; Hermann et al. 2011), and by others as synonymous with ‘ecosystem services’ (De Groot et al. 2002). Ecosystem processes are widely seen as the dynamic interactions among biotic and abiotic elements of ecosystems and are often quantified and referred to in terms of the flow of materials and energy over time. When an ecosystem process has a direct positive effect on human health or economic well-being, it becomes what is often called an ecosystem service. A core ecosystem service addressed by the WFD is water services (cf. Article 9, Article 2(38), and Annex III), but ecosystem function also includes providing benefits to all other living species in ecosystems.

The Marine Strategy Framework Directive^3^ (MFSD) of 2008, which is even more explicitly based on an ecosystem approach than the WFD, includes the concept of ecosystem processes in its definition of environmental status (cf. article 3(4)). Structure, function, and, with reference to the MSFD, processes, are key concepts used to identify and describe an ecosystem.

Blew (1996) mentions that ecosystems may be defined in various ways that focus on species, abiotic elements, or geographic units. With reference to Tansley (1935), Blew suggested that ecosystems may be defined by the interplay among species, and abiotic resources and conditions, and this definition fits nicely with the concept of processes, and also links to structures and functions.

The WFD applies an indicator-based ecosystem approach to managing aquatic ecosystems. The description of complex aquatic ecosystems is broken down into a measurable, indicator-based description of selected elements of the ecosystem: biological quality elements. In practice, this is put into operation by the Member States’ selection of indicators that are suitable for describing the state of the quality elements, ranging from an ecosystem with poor status to an ecosystem with high status. The more closely these indicators are related to the overarching ecosystem’s structures and processes, the better suited the legal and administrative framework will be to achieve the Directive’s goal of protecting the ecosystems.

Although Member States have the freedom to decide which components, structures, and processes should be addressed when identifying and describing terrestrial ecosystems that depend on groundwater, these must be closely aligned with the establishment of relevant indicators for determining damage to the ecosystem.

### The relevant scale of WFD terrestrial ecosystems

The WFD provides a rather broad framework for the Member States’ identification of water entities: bodies of ground- and surfacewater, for which environmental targets are set, sub-basins, river basins, and finally, river basin districts, for which programmes of measures (PoMs) are set. But there is no framework for determining the relevant spatial scale for identifying a groundwater-dependent terrestrial ecosystem.

Annex II of the Directive includes some size categories that Member States may apply, but do not have to, when they classify bodies of water. These are very broad categories. Streams and rivers may be divided by type into bodies that cover 10 km^2^ to over 10,000 km^2^ catchment areas, whereas the size parameters for lakes range from 5 km^2^ to over 100 km^2^.

The question of scale also arises when it comes to terrestrial ecosystems. Habitats such as springs and groundwater-dependent bogs, mires, and fens often cover a small area, of less than 0,01km^2^ to perhaps 2 km^2^. An area with such a habitat may be described in terms of its species, structures, and processes. However, an aquifer or a body of groundwater will typically feed a network of such wetland habitats distributed along a coast or stream valley, and their connected species, and such a network could also be considered an ecosystem. Even an entire valley could be described as a terrestrial groundwater-dependent ecosystem. So, ecologically and legally, what scale is most appropriate?

The presence of aquifers is dependent on the geological substrate (Wendland et al. 2008). Various types of sediments and rocks may form aquifers, most notably porous sediments, such as sand and gravel, but also fractured formations of rocks and limestone. These hydrogeological properties of soil and subsoil are the outcome of the geological processes that created the physical landscape. Together, geological events and processes have determined the composition of the soil and rocks, and the morphology of the landscape, and have established the premises for associated groundwater-dependent habitat types (Wheeler 1980a, b, c). Thus, groundwater-dependent ecosystems may be defined geographically at the landscape-level, based on specific populations and networks of groundwater-dependent habitats. In that case, a groundwater-dependent terrestrial ecosystem will comprise several areas, that are jointly fed by one or more bodies of groundwater and assessed as serving as habitats for populations of plants and animals, in which species and the environment interacts through ecological processes.

Europe has undergone losses comparable to 80% of its historical wetlands, and most remaining terrestrial wetlands will be modified or impacted by water loss or eutrophication (Verhoeven 2014). Sechu et al. (2023) have documented a substantial decline in Denmark’s groundwater-dependent terrestrial ecosystems with an estimated loss of meadow and bog/fen-area of 65–90% compared to the beginning of the nineteenth century. Schoukens (2022) reports this same sort of development in Flanders.

Originally, the European landscapes had continuous and widely distributed groundwater-dependent ecosystems, but these have gradually been fragmented by drainage and cultivation. However, the scattered remnants of those may still be regarded as groundwater-dependent ecosystems.

All processes and flow paths occur at different spatial scales, and the choice of the most appropriate spatial scale should support the relevant management decisions (Skånes 1997). As discussed in CIS document no. 34,^4^ using a landscape-scale approach and managing water balances for very large catchment areas may mask the variability of bodies of groundwater and the water demands of a landscape, and ultimately hide crucial pressures and impacts. However, if each of the persisting small, undrained, and uncultivated remnants of bogs, meadows and other groundwater-dependent habitats should be assessed, and managed, as an independent or separate ecosystem, much more detailed knowledge of the local interconnections between aquifers and the remnants of terrestrial nature will be needed. Also, landscape-scale ecosystems will better align with the scale of bodies of groundwater, and will typically include a whole range of habitats in various ecological conditions. The landscape, or land unit (Zonneweld 1989), as the entity for environmental protection and management is commonly suggested (van Buuren 1991; Arts et al. 2017). Correspondingly Josefsson (2014) has argued for the landscape-scale management of ecosystems covered by the WFD.

Alternatively, a habitat-scale approach—such as the approach of the Habitats Directive—would emphasize the relicts of habitats in the modern landscape. Apart from conflicting with the stated emphasis of WFD on an ecosystem approach, this limited scale would also complicate the causal linking of huge ground water bodies with small habitats. Further, it would overlook the massive degradation reflected by the historical losses and thereby also the potential for future restoration—even if such restoration is not mandatory.

### The question of groundwater dependency

All abstraction of water for human use will alter the natural water cycle. It is not possible to abstract water without that water missing somewhere else in the ecosystem. The purpose of the WFD is not to protect groundwater as such from abstraction or drainage, but to protect groundwater-dependent ecosystems. That is why the status of ground water bodies affected by abstraction and drainage is assessed by its effect on the affected ecosystems. It follows first of all from Annex V, section 2.1 where good quantitative status of a groundwater body requires that there is no anthropogenic alteration of the ground water level preventing the achievement of the environmental objectives for surface water or causing significant damage to terrestrial ecosystems dependent the groundwater body, but also from the definition of available groundwater resource in Article 2(27) that is linked to the potential damage from abstraction on aquatic and terrestrial ecosystems.

The WFD thus requires the identification of interconnections between bodies of groundwater and terrestrial ecosystems prior to assessing whether the ecosystem’s needs are being met. It follows from Annex II, section 2.1 (cf. Article 5) that, as part of the initial characterization of all groundwater, regarding its use and the risk of its not meeting the objectives, Member States must identify the bodies of groundwater on which terrestrial ecosystems are directly dependent. If the environmental objectives of the bodies of groundwater are at risk of not being met, it follows from Annex II, section 2.2 that for each body of groundwater there must be an overview of the terrestrial ecosystems with which the body of groundwater is connected, and an estimate of the water exchange with them.

According to the definition in Table 2.1.2 of Annex V, good quantitative status of a body of groundwater requires that the level of groundwater is not subject to anthropogenic alterations that will result in any significant damage to terrestrial ecosystems, *which depend directly on the body of groundwater*. The same wording is used with regard to good chemical status. The terrestrial ecosystems covered by the Directive are generally described as *directly dependent*,^5^*associated*,^6^ and *dynamically linked to*^7^ bodies of water or aquatic ecosystems.

The relationship between groundwater and ecosystems may be direct, in the sense that water seeps through the root zone, as is the case of springs, fens, and minerotrophic mires, where not only the water saturation, but also the transport and discharge of minerals is important for habitat functioning (Wassen et al. 2005). But the impact may also be indirect, meaning a groundwater level below the root zone that sustains the secondary water table, for example, that of an ombrotrophic bog, or that contributes to the upward movement of water in the case of a prolonged drought.

In sense conveyed in the Directive’s wording, a terrestrial ecosystem could possibly be ‘associated with’ or ‘dynamically linked to’ a body of groundwater, without being ‘directly dependent’ on the body of groundwater, and thereby being relevant to the assessment of the status of the body of groundwater. However, that seems unlikely. There are no indications that the use of various terms in the Directive's motivation, purpose, definitions, and provisions for characterization regarding the connection between bodies of groundwater and terrestrial ecosystems has any intent. The essential point for determining this relationship must be that anthropogenic changes in the water level or in the chemical composition of groundwater will lead to significant deterioration of the biological status of the ecosystem.

Terrestrial habitats that depend on groundwater include mires, bogs, fens, and swamps, but possibly also meadows and wet heaths. Although some of these are described as ombrotrophic, meaning that they receive only rainwater, a bog may still originate from a groundwater-fed lake, and a lowering of the groundwater table below the bog may lead to bog desiccation, encroachment of woody species, and eventual ecosystem collapse (Terasmaa et al. 2020). Even in grassland, dune, and heath ecosystems, fauna may depend on high groundwater tables, particularly on sandy soils where the availability of groundwater prevents the severe consequences of summer droughts (e.g. Örvössy et al. 2013). Thus, terrestrial ecosystems may differ in the degree and timing of their dependency on groundwater recharge or a high groundwater table, and in some ecosystems, the dependency will be measurable only during periods of prolonged drought (Murray et al. 2003).

A number of habitats listed in Annex I of the Habitats Directive^8^ are generally dependent on groundwater. These comprise bog, mire, fen, meadow, wet heath, dune depression, salt marsh, and forest swamp habitats. However, the Annex I list in the Habitats Directive cannot be considered exhaustive for groundwater-dependent habitats, as wetlands with willow and birch forest, or reed swamps and tall herb fens, may also be groundwater-dependent, and occur in a mosaic with other wetland habitats as part of larger groundwater-dependent ecosystems (Wheeler 1980a, b, c). Also, grassland, heath, and forest habitats may periodically depend on a high, stable groundwater level. Favourable conservation status for Annex I habitats refers to distribution, area, structure and function and future prospects. Even though ground water availability is not specifically mentioned, it is inevitably linked to the indicators of structure and functions of e.g. alkaline fens and springs (e.g. Andersen et al. 2013).

## What should be considered ‘significant impact’?

### ‘Significant impact’ in EU law

The protection offered to groundwater-dependent terrestrial ecosystems under the WFD is embedded in the notion that there should be no ‘significant damage’ to a GWDTE as a result of anthropogenic changes to the groundwater table or concentrations of pollutants.

There is, however, no further clarification in the WFD when it comes to the criterion of ‘significant damage’ in relation to groundwater-dependent terrestrial ecosystems. In this respect, other EU legislation, most prominently the Habitats Directive, but also the Environmental Liability Directive can be relevant.

The WFD mainly uses ‘deterioration’ as the general criterion in Article 4 for both surface and groundwater bodies. With the Weser judgment,^9^ the CJEU established that any decline in status class for a single quality element constitutes deterioration in the sense of WFD, even if it does not result in a fall in the classification of the body of surface water as a whole. The indicator-based description of aquatic ecosystems established by the Directive, and the need for intercalibration to ensure comparability among Member States, has formed the basis for this narrow margin for ‘deterioration’ or what should be perceived as a negative change in status of a body of water. Thus, not every measurable decrease in the indicator-based values constitutes deterioration, only if an individual quality element is already in the lowest class, any decline constitutes a ‘deterioration of the status’ of a body of surface water.^10^

The CJEU has taken a somewhat similar, or perhaps even stricter, approach to chemical groundwater status. In case C-535/18 on the discharge or infiltration of rainwater run-off from a road, the Court stated that ‘the exceedance, in a body of groundwater, of a single one of the quality standards or threshold values, within the meaning of Article 3(1) of Directive 2006/118, must be classified as amounting to an infringement of the obligation to prevent the deterioration of the status of a body of groundwater.^11^ Likewise, ‘any subsequent increase in the concentration of a pollutant that, with reference to Article 3(1) of Directive 2006/118, already exceeds an environmental quality requirement or a limit value set by the Member States also constitutes a deterioration.’^12^ The Court also noted that the failure to comply with a quality element at a single monitoring point should be regarded as a deterioration of the chemical status of the body of groundwater.^13^

Regarding quantitative status, the Court has established that deterioration of quantitative groundwater status within the meaning of WFD presupposes an increased overexploitation as compared with an earlier situation.^14^ This implies, that if the body of groundwater is already of poor quantitative status, further increase in abstraction from that body of groundwater will constitute deterioration in the sense of Article 4(1)(b)(i). In addition, the Court stated that addressing existing overexploitation falls under the obligation to enhance or restore water bodies in Article 4(1)(b)(ii).^15^ The Court found that in the specific case there was a failure under WFD Art. 11 to take appropriate measures to prevent disturbance of the protected habitat types in a Natura 2000 site caused by the abstraction of groundwater^16^ and that the continued disturbance of Natura 2000 habitats caused by existing abstraction of groundwater violated Article 6(2) of the Habitats Directive.^17^

Turning to the Habitats Directive, a number of court cases illustrate what constitutes significant effect or ‘adverse effect’ on terrestrial ecosystem components, or ecosystems as such. In the case C-404/09 regarding mining in an area with habitats for the priority species Cantabrian brown bear and Cantabrian capercaillie, the court found that the loss of a forested transit route, and disturbance by noise and vibration caused by mining operations had resulted in habitat loss. The habitat loss made it more difficult for the bears and capercaillies to roam, and this had the potential to affect the populations in the area to the extent that there was the risk of them being fragmented into two subpopulations, or even separate populations.^18^ Spain argued that this loss of habitat was unimportant for the overall conservation of the species, since the areas affected did not contain any breeding or rearing ground. That argument was not accepted by the court. Even though the areas may not have contained breeding or rearing ground, they could conceivably serve as habitats for purposes other than breeding or rearing, such as living, hibernating or roaming^19^; regarding the capercaillie, if the areas in question had not been lost as habitats, the possibility they could have become breeding ground could not be excluded.^20^

The CJEU has similarly established quite strict criteria for determining when a Natura 2000 site is adversely affected regarding its nature types, or rather, when it may be ruled out that a site is damaged. In the case C-258/11, the Court stated that a site’s integrity is adversely affected if a project or plan is ‘liable to prevent the lasting preservation of the constitutive characteristics of the site concerned that are connected to the presence of a natural habitat type’.^21^ The case involved the permanent destruction of 1.47 ha of an approximately 270 ha limestone plateau, which was a priority habitat type, but the Court did not take a direct position on whether this constituted damage in this specific case, as this had to be settled by the national court according to the criteria established by the CJEU.

The Environmental Liability Directive (ELD) aims to establish a framework for implementing preventive or remedial measures in the event of harmful actions. Article 2(1)b refers to ‘water damage’ as ‘any damage that significantly adversely affects the ecological, chemical or quantitative status or the ecological potential, as defined in Directive 2000/60/EC, of the waters concerned’. With regard to ‘water damage’, where WFD quality elements and indicator-based descriptions of aquatic ecosystems have paved the way for a detailed status assessment, the Commission Guidance stipulates that emphasis must be on whether there is measurable permanent or temporary loss or deterioration of a status element, so that the affected water no longer exhibits its relevant characteristics for this status element.^22^ Josefsson (2018) takes this a step further and suggests that damage of waters is significant ‘when all the quality elements used to classify a body of water, according to Annex V, deteriorate by one class; when a body of water is affected by deterioration that results in a class change for the biological quality elements not in lowest class, and a measurable change in the quality element in a lowest class; and when all the biological quality elements in a body of water are in a lowest class deteriorate measurably and the deterioration of that body of water compromises the achievement of WFD objectives for other bodies of water’.

With regard to damage to protected species and natural habitats, Annex I of the Directive, referenced in Article 2(1)(a), provides that the objective of reaching or maintaining favourable conservation status is the point of reference to which damage must be considered. According to Annex I, significance should be determined by measurable data, such as the number of individuals, their density or the area they cover, the role of the particular individuals or of the damaged area in relation to the species or to habitat conservation, the rarity of the species or habitat (assessed at local, regional, and higher levels, including community level), the species’ capacity for propagation, their viability or the habitat’s capacity for natural regeneration, and the species’ or habitat’s capacity for recovery. In case C-297/19, the CJEU confirmed that only damage that has a certain degree of seriousness may be considered damage to protected species and natural habitats.^23^ Similarly, Annex I signals that some damage or instances are not significant, such as normal ecosystem processes that are not initiated or driven by human intervention, and those driven by human intervention that either result in changes to the ecosystem that occur as a result of normal site or habitat management, or cause variations that are smaller than natural fluctuations.

The criteria stipulated in Annex I for assessing significant damage to protected species and natural habitats, to which the CJEU also refers, reference core ecosystem properties, and it is apparent from case law that the CJEU consolidates an ecosystem approach regarding the protection of species and habitats, when applying the provisions of both the Habitats Directive and the Environmental Liability Directive. It is probable that the CJEU’s strict interpretation could also prevail, regarding significant damage to terrestrial ecosystems protected by the WFD, and that adverse effects to the long-term preservation of constitutive characteristics of a groundwater-dependent habitat would qualify as significant damage.

The challenge is to establish a sufficiently clear causal link between damaged habitats or ecosystems, and a body of groundwater. For that reason, the Member States’ choice of indicators that are sensitive to lowering of the groundwater table or pollution of the body of water remains extremely important.

Under Article 6(3) of the Habitats Directive, the assessment of implications for a protected site must be identified in the light of the best scientific knowledge in the field, and an activity only authorized if it is made certain that it will—with no reasonable scientific doubt—not adversely affect the integrity of the site.^24^ There are no indications whether this ‘burden of proof’ will also be applied in case of damage on a terrestrial ecosystem due to pollution or scarcity of groundwater.

### Scope of protection: Impact of what?

The identified groundwater-dependent terrestrial ecosystems are subject to an indirect protection based on the quantitative and chemical status of bodies of groundwater.

Terrestrial ecosystems are protected against the impact of *water pollution* by being linked to the definition of pollution. Among other things, pollution is the discharge of substances that may damage the quality of terrestrial ecosystems that are directly dependent on water ecosystems (cf. Article 2, no. 33). When it comes to the chemical status of bodies of groundwater, good chemical status requires that the groundwater’s level of pollutants to not cause significant damage to the terrestrial ecosystems that are directly dependent on that body of groundwater (cf. Article 2, no. 25 and Annex V Sect. 2.3.2). The Groundwater Directive lays down more detailed quality standards for certain substances, and, more importantly, Article 3 of the Directive requires the Member States to set specific threshold values that consider the impact on, and interrelationships with, associated terrestrial ecosystems. The legal implications of the threshold values do not emerge clearly from the Directive. Yet, according to the CJEU, the threshold values, together with quality parameters of the Directive, constitute a quality element for the purpose of determining the chemical status of a groundwater body, and accordingly the CJEU classifies exceedance of threshold values as deterioration.^25^

From Article 2, no. 28, with reference to Annex V, Table 2.1.2, it follows that terrestrial ecosystems are protected against the impact of anthropogenic alterations to the groundwater level. The definition in Table 2.1.2 explicitly refers to the abstraction of groundwater, and the Directive’s definition of ‘available groundwater resource’ (cf. Article 2, no. 27) states that the extraction of groundwater must not cause significant damage to associated terrestrial ecosystems. From Article 4 (1)(b)(ii), it also follows that Member States must ensure a balance between extraction and recharge of bodies of groundwater as part of the obligation to achieve good groundwater status.

These references to abstraction of groundwater could lead to the conclusion that only the impact on the water table due to the abstraction of groundwater should be considered, when assessing whether there is a significant impact on a groundwater-dependent terrestrial ecosystem resulting from water scarcity. CIS guideline no. 12 (2003) on wetlands^26^ seems to take that point of view. This states that these provisions are ‘not designed to protect terrestrial ecosystems directly dependent on bodies of groundwater from other sources of damage, for example: drainage’. However, this is not the case. If the achievement of good status for a body of groundwater is considered to be at risk, Annex II, section 2.3 requires the Member States to assess the impact of human activity, including *land use in the catchment*, *anthropogenic alterations to the recharge characteristics such as rainwater and run-off and drainage*. Accordingly, drainage is one of the human activities listed in Annex II section 2.4 that may justify setting lower objectives for a body of groundwater, due to changes in groundwater levels.

Legal scholars have criticized the CIS guidelines (Josefsson 2015), and they have been dismissed by the CJEU when insufficiently derived from the Directive.^27^ It seems beyond reasonable doubt that *both* drainage that lowers the water table of groundwater-dependent habitats *and* drainage that affects the recharge of a body of groundwater should be taken into account, when assessing the human impact on a body of groundwater and its associated terrestrial ecosystems.

## Pitfalls and management options

The WFD allows the Member States a certain margin of discretion with regard to determining relevant components, structures, and processes, when identifying and describing groundwater-dependent terrestrial ecosystems. Yet, the Directive’s underlying ecosystem approach suggests that a narrow, fragmented approach, based on small patches of residual nature is inappropriate. Furthermore, in practice, in many cases it is impossible to directly link the occurrence and quality of a local patch of mire or meadow to a specific aquifer. The actual knowledge of underground layers is limited, and the designation of aquifers and bodies of groundwater is generally based on conceptual models, many of which involve a great deal of uncertainty: the smaller scale, the greater the uncertainty. That is why we argue that designating terrestrial ecosystems at the landscape-level is better suited to meeting the Directive’s purpose, and is the basis for adequate water management and regulation. As explained below landscape-level designation of terrestrial ecosystems may facilitate the use of relevant regulatory measures for identifying new of existing harmful activities, including the determination of causal effects and significant damage, but also for identifying appropriate restoration measures, including the possible reestablishment of a groundwater level, able to sustain groundwater-dependent ecosystems.

The regulatory options for addressing activities that may harm groundwater-dependent terrestrial ecosystems rely on both the establishment of a causal link between activities and harm and assessing the level of ‘significant impact’. Both aspects are complex and difficult to determine in practice, and even more so if a narrow, fragmented approach to GWDTE’s is applied: For example, whether new water abstraction will affect the groundwater level in such a way as to cause a significant harm. Neither is the legal concept of significant impact well-defined, as it cuts across various concepts addressed in the EU Water Framework Directive and the Habitats Directive. Under the WFD, the CJEU appears to rely on a concept of ‘deterioration’ that is based on detailed, and primarily quantitative, criteria that rely on the established categories and quality elements. However, deterioration of water status in WFD is not necessarily the same as significant damage in ELD.

The diversity and number of habitats and species protected by the Habitats Directive makes an intercalibrated system intractable. Instead, the CJEU, and also the Commission,^28^ have applied an ecosystem approach, based on whether the Natura 2000 sites in question can maintain the structure and processes that are important for preserving the habitats and species that the site have been designated to protect. If the special structure and functions of the site that are necessary to sustain the habitats or species over the long-term are reduced, this constitutes deterioration. This holistic ecosystem approach is replicated in the CJEU’s application of the Environmental Liability Directive. When determining significant damage to groundwater-dependent terrestrial ecosystems, it is probable that the Habitats Directive’s threshold for significant damage and the ELD’s assessment criteria will prevail. This ecosystem perspective in determining significant damage also calls for a landscape-level approach to the designation of GWDTE’s.

A particular pitfall in the foregoing process, which can impair the WFD’s protection of ecosystems, relates to existing harmful activities. It is linked to the definition of groundwater in article 2(2). Groundwater tables and flows are lowered by water abstraction and by drainage. These are the main pressures. Both pressures may have a significant impact on groundwater-dependent nature and thus have a significant impact on the ecosystem. However, when aquifers are already reduced by drainage or abstraction, when compared to their natural state, Member States may, like Denmark do, deliberately designate the individual aquifers and bodies of groundwater in accordance with their *already lowered* groundwater table. This is done with reference to the definition of groundwater in article 2(2), as water below the surface of the ground and *in the saturation zone*, despite the unsaturated zone in the topsoil being extended, compared to the natural state.

There is no obligation under the WFD to restore groundwater-dependent terrestrial ecosystems to the states they had before the adoption of the WFD; the same cannot be concluded regarding the states of bodies of groundwater and aquifers. Unlike the determination of potential ‘deterioration’ or ‘significant damage’, Member States’ general obligation to protect, enhance and restore with the purpose to achieve good quantitative (and chemical) status means that measures should be taken to put an end to groundwater abstraction or drainage with a significant impact on terrestrial ecosystems, unless it may fall under the derogation clauses. Unless the derogation conditions are met, and they are accounted for in the river basin management plan, lowering the groundwater table of aquifers—by drainage or abstraction—that has resulted in, or at present results in, significant damage to the structure and the functioning of the ecosystem must be addressed. In other words, the WFD imposes the obligation to restore the hydrological conditions necessary for intact terrestrial ecosystems dependent on groundwater, but not the terrestrial ecosystems, as such. Yet, the 2024 EU Nature Restoration Regulation^29^ sets specific requirements for the restoration of listed habitat types that are not in good condition, including e.g. alkaline fens.

A wide range of habitats may depend on high levels of groundwater over the long term; therefore, a narrow approach that identifies only residual patches of few selected nature types as ‘ecosystems’ will not meet the Directive’s purpose, as it is stated in article 1. Instead, designating and characterizing the structure and function of groundwater-dependent ecosystems at the level of hydrological landscapes may reduce uncertainty regarding causal effects and significant damage and emphasize the need to address existing harmful activities—such as drainage and excessive water abstraction—to achieve good groundwater status, also with regard to groundwater-dependent ecosystems. A landscape perspective is not only compliant with the legislation but also a scientifically appropriate scale to describing the historical man-made changes of the natural hydrological system as well as the future restoration potential.

## Footnotes

1 European Parliament and Council Directive 2000/60/EC, establishing a framework for Community action in the field of water policy [2000] OJ L327/1.

2 Council Directive 76/464/EEC on pollution caused by certain dangerous substances discharged into the Community’s aquatic environment. Council Directive 78/659/EEC of 18 July 1978 on the quality of fresh waters needing protection or improvement in order to support fish life. Council Directive 79/923/EEC of 30 October 1979 on the quality required of shellfish waters. Council Directive 91/271/EEC of 21 May 1991 concerning urban wastewater treatment. Council Directive 91/676/EEC of 12 December 1991 concerning the protection of waters against pollution caused by nitrates from agricultural sources.

3 Directive 2008/56/EC of the European Parliament and of the Council of 17 June 2008, establishing a framework for community action in the field of marine environmental policy.

4 Guidance document concerning the application of water balance for supporting the implementation of the WFD. Final—Version 6.1—18/05/2015.

5 Recital 23 of the preamble, Article 1(a), Annex II Sect. 2.1., Annex V Sect. 2.1.2., Annex V Sect. 2.3.2.

6 Recitals 20 of the preamble, Article 1 no. 27, Annex II Sect. 2.4.

7 Annex II, point 2.2.

8 Council Directive 92/43/EEC of 21 May 1992 on the conservation of natural habitats and of wild fauna and flora.

9 C-461/13 Bund für Umwelt und Naturschutz Deutschland eV (Weser), ECLI:EU:C:2015:433.

10 Ibid. para. 70.

11 C-535/18 Land Nordrhein-Westfalen, ECLI:EU:C:2020:391, para. 109. See further below 3.2.

12 Ibid. para. 110.

13 Ibid. para. 118.

14 C-559/19 Commission v Spain (Donana), ECLI:EU:C:2021:51, para. 49.

15 Ibid.

16 Ibid. para. 141.

17 Ibid, para. 169–172.

18 C-404/09 Commission v Spain, ECLI:EU:C:2011:768, para 148 + 189–191.

19 Ibid. paras 133 and 188.

20 Ibid. paras 131–134.

21 C-258/11 Sweetman and others, ECLI:EU:C:2013:220, para. 48.

22 Communication from the Commission. Guidelines for a common understanding of the concept of ‘environmental damage’ as defined in Article 2 of Directive 2004/35/EC of the European Parliament and of the Council on environmental liability regarding the prevention and remediation of environmental damage, 2021/C 118/01, point 169.

23 Case C-297/19, Naturschutzbund Deutschland—Landesverband Schleswig–Holstein eV, ECLI:EU:C:2020:533, para. 34.

24 Case C-127/02 Waddenzee, ECLI:EU:C:2004:482, para. 61.

25 Case C-535/18, Land Nordrhein-Westfalen, ECLI:EU:C:2020:391, para. 107–109.

26 Common Implementation Strategy for the Water Framework Directive (2000/60/EC), Guidance document no. 12, The role of wetlands in the Water Framework Directive, 2003, p. 22.

27 C-525/20, Association France Nature Environnement, ECLI:EU:C:2022:350, para 30 and 31.

28 The Commission’s guidance, p. 30.

29 Regulation 2024/1991 of the European Parliament and of the Council of 24 June 2024 on nature restoration and amending Regulation (EU) 2022/869.
